# Functional traits and drought strategy predict leaf thermal tolerance

**DOI:** 10.1093/conphys/coad085

**Published:** 2023-11-13

**Authors:** Justin M Valliere, Kekoa C Nelson, Marco Castañeda Martinez

**Affiliations:** Department of Plant Sciences, University of California Davis, One Shields Ave., Davis, CA 95616, USA; Department of Biology, California State University Dominguez Hills, 1000 E Victoria St., Carson, CA 90747, USA; Department of Biology, California State University Dominguez Hills, 1000 E Victoria St., Carson, CA 90747, USA; Department of Biology, California State University Dominguez Hills, 1000 E Victoria St., Carson, CA 90747, USA

**Keywords:** Chlorophyll fluorescence, climate change, ecophysiology, functional traits, heat stress, Mediterranean ecosystems, photosynthesis, temperature response curves

## Abstract

Heat stress imposes an important physiological constraint on native plant species—one that will only worsen with human-caused climate change. Indeed, rising temperatures have already contributed to large-scale plant mortality events across the globe. These impacts may be especially severe in cities, where the urban heat island effect amplifies climate warming. Understanding how plant species will respond physiologically to rising temperatures and how these responses differ among plant functional groups is critical for predicting future biodiversity scenarios and making informed land management decisions. In this study, we evaluated the effects of elevated temperatures on a functionally and taxonomically diverse group of woody native plant species in a restored urban nature preserve in southern California using measurements of chlorophyll fluorescence as an indicator of leaf thermotolerance. Our aim was to determine if species’ traits and drought strategies could serve as useful predictors of thermotolerance. We found that leaf thermotolerance differed among species with contrasting drought strategies, and several leaf-level functional traits were significant predictors of thermotolerance thresholds. Drought deciduous species with high specific leaf area, high rates of transpiration and low water use efficiency were the most susceptible to heat damage, while evergreen species with sclerophyllous leaves, high relative water content and high water use efficiency maintained photosynthetic function at higher temperatures. While these native shrubs and trees are physiologically equipped to withstand relatively high temperatures in this Mediterranean-type climate, hotter conditions imposed by climate change and urbanization may exceed the tolerance thresholds of many species. We show that leaf functional traits and plant drought strategies may serve as useful indicators of species’ vulnerabilities to climate change, and this information can be used to guide restoration and conservation in a warmer world.

## Teaser

Rising temperatures pose a major threat to native plant species, particularly in urban areas. We evaluated how native plants in a restored urban nature preserve responded to experimental heat stress. We found that species differed in levels of heat tolerance and that these differences can be explained by leaf traits.

## Introduction

Rising global temperatures and increasingly severe heat wave events due to human-caused climate change pose a major threat to the conservation of biodiversity (Malcolm *et al.,* 2006; Perkins-Kirkpatrick and Lewis, 2020). In the coming years and decades, species throughout the world could face extinction if they are unable to adapt or acclimate to new temperature extremes or shift their ranges to more suitable climates (Malcolm *et al.,* 2006; Parmesan, 2006). As sessile organisms, plants may be particularly vulnerable to rapid changes in climate. While plant species have evolved a variety of morphological, physiological, biochemical and phenological traits to cope with heat stress (Wahid *et al.,* 2007), elevated temperatures in the Anthropocene will likely threaten the persistence of many taxa in the not-so-distant future. Indeed, climate warming has already contributed to large-scale plant mortality events across the globe (Anderegg *et al.,* 2013; Hammond *et al.,* 2022). Despite this risk, the vulnerability of plants to extreme heat events associated with climate change is underappreciated and understudied (Breshears *et al.,* 2021). A greater understanding of thermal tolerance thresholds and underlying plant functional traits is necessary if we are to implement appropriate interventions to conserve plant diversity in a warmer world.

Elevated temperatures associated with climate change will be further amplified by urbanization (Kalnay and Cai, 2003). Cities typically experience higher temperatures than outlying areas due to the lack of vegetation, low albedo and high density of buildings and pavement that absorb and retain heat (Ramamurthy and Sangobanwo, 2016). This urban heat island effect results in higher mean temperatures overall within cities, but it may also exacerbate extreme heat wave events (Zhao *et al.,* 2018). Plants in urban areas are therefore especially vulnerable to rising temperatures, in addition to other stressors such as atmospheric pollution and drought (Grimm *et al.,* 2008; Esperon-Rodriguez *et al.,* 2022; Marchin *et al.,* 2022). This poses a significant risk to vegetation within cities, as well as the key ecosystem services that urban greenspaces provision (Breuste *et al.,* 2013), including the ability of plants to directly combat urban warming. It also severely complicates efforts to conserve and restore native plant species within highly urbanized areas, which is an increasingly important strategy for protecting biodiversity in the Anthropocene (Dearborn and Kark, 2010; Segar *et al.,* 2022).

**Figure 1:**
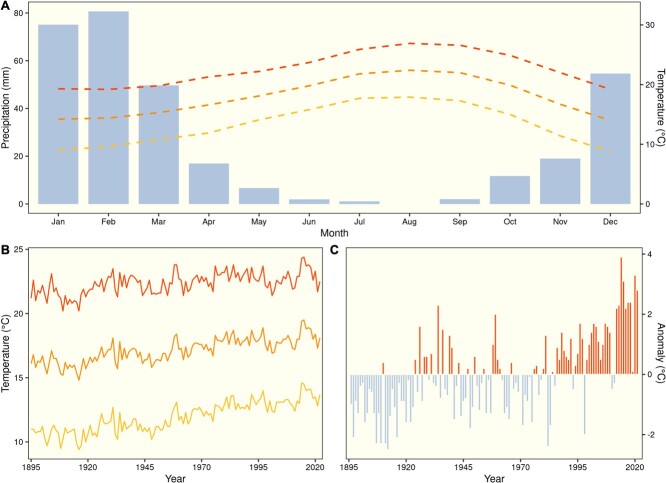
Typical annual climate patterns of the study site in Carson, CA, USA (A), including average monthly precipitation (blue bars) and average minimum (yellow), mean (orange) and maximum (red) temperatures by month. Also shown are average annual temperatures from 1895 to 2022 for the study site (B), including minimum (yellow), mean (orange) and maximum (red) temperatures, as well as temperature anomalies (deviations from long-term averages) for the state of California during the same period (C).

Biodiversity hotspots of the world’s Mediterranean ecoregions may also be especially at risk under accelerating climate change and other anthropogenic disturbances (Malcolm *et al.,* 2006; Matesanz and Valladares, 2014), including urbanization (McDonald *et al.,* 2013). In California, temperatures have steadily risen over the past hundred years (Fig. 1) and are projected to increase anywhere from 2 to 7°C (relative to historical temperatures between 1950 and 2005) by the end of the century depending on greenhouse gas emissions (Pierce *et al.,* 2018). These temperature increases will be exacerbated in urban areas. For example, in Los Angeles—one of the largest megacities in the world—the urban heat island effect is especially pronounced (Vahmani and Ban-Weiss, 2016), with temperatures up to 5–9°C warmer in urbanized areas (Dean, 2015). This threatens the very limited amount of already imperiled native plant diversity that remains within the city (Schwartz *et al.,* 2006; Avolio *et al.,* 2020). In such a developed urban landscape, the few opportunities that exist for preserving native vegetation include protecting and restoring small reserves within the city and encouraging the use of native plant material in landscaping (Longcore, 2006). For these approaches to be viable in the long term, however, the species targeted for such efforts must be able to withstand the novel and stressful abiotic conditions that exists within the urban matrix.

In this study, we evaluated the impact of elevated temperatures on a group of functionally and taxonomically diverse native plant species found in a restored urban nature preserve in southern California using measurements of chlorophyll fluorescence as an indicator of leaf thermotolerance. Chlorophyll fluorescence—specifically the ratio of variable to maximum chlorophyll fluorescence (*Fv/F_m_*), or the maximum quantum yield of photosystem II (PSII)—is a useful proxy for evaluating photosynthetic efficiency and levels of plant stress (Schulze and Caldwell, 2012). Because PSII is highly sensitive to heat stress, *Fv/F_m_* values have been used extensively to evaluate thermotolerance in plants (Maxwell and Johnson, 2000; O'Sullivan *et al.,* 2017; Sastry *et al.,* 2018). Our aim was to determine if species’ traits and drought strategies (i.e. drought deciduous species that avoid seasonal drought versus evergreen species that remain active year-round) could serve as useful predictors of thermotolerance and provide insight into the ability of these native species to withstand elevated temperatures under increasing climate warming and as a result of the urban heat island effect. We predicted that drought deciduous species with short-lived leaves designed for rapid carbon gain and water use would exhibit lower levels of leaf thermotolerance compared with evergreen species with longer lived sclerophyllous leaves (i.e. tough and dense leaves) and more conservative water use. For example, evergreen species that remain physiologically active during the hot summer months might be expected to possess leaf-level adaptations for withstanding higher temperatures compared with drought deciduous species that drop their leaves during the hottest and driest periods of the year.

## Materials and Methods

### Study species

**Figure 2:**
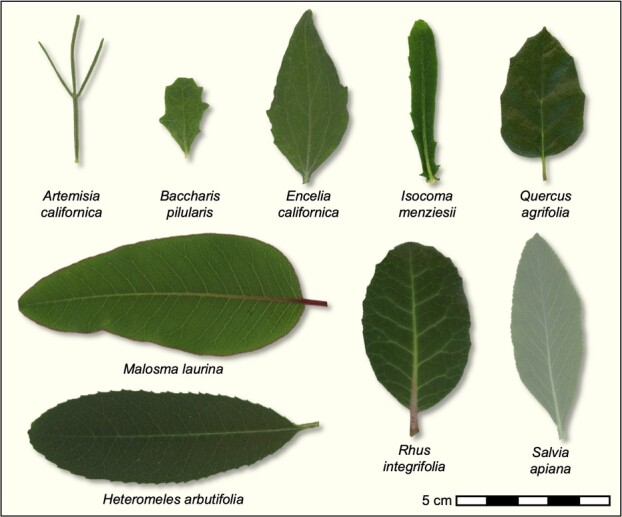
Images of representative leaves of each species included in the study.

We selected nine shrub and tree species that are native to southern California that vary in leaf morphology and drought strategy (Table 1, Fig. 2). These included woody species from a number of plant families that commonly occur in coastal sage scrub, chaparral, and oak woodlands in the region. These species possess a diversity of morphological, phenological and physiological traits and water use strategies (Harrison *et al.,* 1971; Poole and Miller, 1975; Ackerly, 2004; Pivovaroff *et al.,* 2018) for coping with seasonal drought in California’s Mediterranean-type climate (Fig. 1A). Several of the selected study species are evergreens, including several chaparral shrubs and a native oak, with sclerophyllous leaves. These species maintain leaves year-round, including during the hot and dry summers of southern California. The other species selected exhibit a drought deciduous strategy in which they produce leaves during winter months when rainfall occurs and then avoid drought by dropping their leaves during the summer. The latter drought strategy is more characteristic of species from coastal sage scrub.

**Table 1:** Native plant species evaluated in the study, including scientific and common names, species codes, plant family and drought strategy

Species	Common name	Family	Drought strategy
*Artemisia californica*	California sagebrush	Asteraceae	Drought deciduous
*Baccharis pilularis*	Coyote brush	Asteraceae	Evergreen
*Encelia californica*	California brittlebush	Asteraceae	Drought deciduous
*Heteromeles arbutifolia*	Toyon	Rosaceae	Evergreen
*Isocoma menziesii*	Coastal goldenbush	Asteraceae	Drought deciduous
*Malosma laurina*	Laurel sumac	Anacardiaceae	Evergreen
*Quercus agrifolia*	Coast live oak	Fagaceae	Evergreen
*Rhus integrifolia*	Lemonade berry	Anacardiaceae	Evergreen
*Salvia apiana*	White sage	Lamiaceae	Drought deciduous

### Study site

This study was conducted on the grounds of California State University Dominguez Hills, in Carson, CA, USA (33.8607, −118.2533). The campus is located in the greater Los Angeles area of southern California and experiences a typical Mediterranean-type climate (Fig. 1A). Due to high levels of urbanization in the region, the study site experiences elevated temperatures as a result of the urban heat island effect (Ramamurthy and Sangobanwo, 2016; [Bibr ref55][Bibr ref55]). All plants utilized in the study were located in the campus’s Heritage Creek Nature Preserve, with the exception of several replicates of *Quercus agrifolia* and *Salvia apiana*, which were sampled from mature individuals located in a nearby native plant garden on campus. The species selected are the most common woody plant species within the preserve and are also representative of coastal sage scrub, chaparral and oak woodland communities in the region. The preserve was restored with local native shrubs, trees and forbs in 2006. It covers an area <0.5 hectares and is surrounded by parking lots, roads and generally unvegetated areas on campus. This study was conducted in late winter and early spring in 2023, which was one of the wettest years on record in southern California, with the site receiving >545 mm of precipitation between December 2022 and March 2023. Rainfall occurred on 50 different days throughout this period, with an average of approximately 5 days between storm events. Thus, the plants used this this study had sufficient and sustained access to soil moisture during the study period.

### Climate data

We acquired climate data for the study site from the PRISM Climate Group at Oregon State University, USA (http://prism.oregonstate.edu). Data are based on a 4-km grid interpolation using source data from multiple weather stations and included 30-year averages for precipitation and mean, minimum and maximum temperature by month (Fig. 1A); annual mean, minimum and maximum temperature from 1895 to 2022 (Fig. 1B); and daily rainfall totals during the study period. We also retrieved annual temperature anomalies (Fig. 1C) for the state of California for the same period from the United States Climate Divisional Database (NOAA, 2023).

### Thermal tolerance assays

In February and March 2023, we tested temperature responses of dark-adapted chlorophyll fluorescence (*Fv/F_m_*) for each of the nine study species. We collected intact, recently matured, sun-exposed leaves from 5 to 10 individuals of each species, depending on the availability of plants within the study site. Prior to conducting assays in the laboratory, collected plant material was placed in buckets with the cut stem or petioles submerged in water and covered with a clear plastic bag. Replicate leaves of each species (*n* = 5) were randomly assigned to one of 18 heat treatments ranging from 24 to 58°C, with 2°C intervals between treatments. Individual leaves were placed in sealed plastic bags with air removed, which were fully submerged for 25 minutes in water baths maintained at the desired temperature. Water baths were heated with Anova Precision Cookers (Anova Applied Electronics, Inc., San Francisco, CA, USA), which heat and circulate water to maintain constant set temperatures. Following heat treatments, plastic bags containing leaf samples were brought to room temperature, placed in paper bags and refrigerated overnight prior to measuring chlorophyll fluorescence. The following day, after leaves had been brought to room temperature, *Fv/F_m_* measurements were taken on dark adapted leaves using OS30p + handheld chlorophyll fluorometer (Opti-Sciences, Inc., Hudson, NH, USA). Measurements were taken in the middle of leaves, away from the mid-rib. For *Artemisia*, which has very fine leaves, we placed clusters of leaves in the leaf clips in order to fully cover the aperture.

### Leaf trait measurements

Using leaves of the same individual plants included in temperature assays, we also collected data on a number of morphological and physiological functional traits. We collected two recently matured leaves from five individual plants for evaluating leaf morphology and water status. We averaged values from the two leaves collected from each plant for subsequent data analysis. We measured leaf thickness (in millimetres) using digital calipers with an accuracy of 0.02 mm, taking two measurements per leaf. We scanned fresh leaves with a digital scanner and measured leaf area using the software ImageJ (Abràmoff *et al.,* 2004). We measured leaf fresh mass (LFM), and then hydrated leaves overnight in water. The following morning, we weighed leaves to obtain rehydrated leaf mass (RLM). We then dried leaves for 48 hours at 70°C in a forced hot air drying oven and again weighed samples to obtain leaf dry mass (LDM). From these data, we calculated specific leaf area (SLA = leaf area/leaf mass; cm^2^ g^−1^), leaf dry matter content (LDMC = (LDM × 1000)/RLM; mg g^−1^), leaf water content (LWC = 1000—LDMC; mg g^−1^), relative water content (RWC = [(LFM—LDM)/(RLM—LDM)] × 100; %), and equivalent water thickness (EWT = (LFM—LDM)/LA; g cm^−2^). We also measured leaf gas exchange on a subset of individuals (*n* = 3) from which leaves were collected using an LI-6800 portable photosynthesis system equipped with a chamber with an internal LED light source and 2-cm^2^ aperture to accommodate smaller leaved species (LI-COR, Lincoln, NE, USA). We took measurements on two recently matured leaves from each individual plant and used the average of these values for subsequent data analysis. Measurements were taken between 10 AM to 12 PM, with photosynthetically active radiation (PAR) set to 1500 μmol m^−2^ s^−1^ and a flow rate of 500 μmol s^−1^. Temperature, CO_2_ concentrations and relative humidity inside the chamber were matched to ambient conditions. Chamber CO_2_ concentration was set at 420 ppm, and temperature and relative humidity were maintained at ~24°C and 65%, respectively. Measurements were taken on a clear day after a precipitation event, when plants had access to adequate soil moisture. Variables measured included carbon assimilation (i.e. photosynthetic rate), stomatal conductance and transpiration. We also used rates of carbon assimilation and transpiration to calculate instantaneous water use efficiency (WUE_i_).

### Thermal tolerance curves

To evaluate the impact of temperature treatments on PSII function and species’ thermal tolerances, we fit four-parameter logistic sigmoid curves to the *Fv/F_m_* values obtained for each species across all temperature treatments using the ‘*drm*’ function in the R package *drc* (Ritz *et al.,* 2015). This was done to obtain species-level estimates of leaf thermotolerance. For each of these models, we set the lower asymptote to zero. We then used these response curves to predict the temperature at which leaves experienced a 50% (*T*_50_) and 95% (*T*_95_) reduction in *Fv/F_m_* relative to the maximum values observed for each individual species using the ‘*ED*’ function. For each species, we also calculated the critical temperature beyond which photosynthetic function is irreversibly damaged (Schreiber and Berry, 1977), or *T*_Crit_, as the point at which the line representing the linear slope of *Fv/F_m_* values surrounding *T*_50_ intersects with the horizontal line representing maximum *Fv/F_m_* values. We then used these obtained thermotolerance values for comparisons with other plant functional traits across study species.

### Statistical analysis

All statistical analysis and graphing was conducted in R Studio Cloud (R version 4.3.0). We first evaluated if plant morphological and physiological functional traits differed by drought strategy of the species included in the study (i.e. drought deciduous or evergreen). To test this, we used individual linear mixed effect models for each trait, with drought strategy included as a fixed effect and species’ identity as a random effect. Models were run using the ‘*lme*’ function in the package *nlme*. We used standard transformations (i.e. log, square root and square functions) as needed to improve normality, and we visually inspected the distribution of model residuals for normality. We used the ‘*aov*’ function to retrieve *F*-statistics and *P*-values for each linear mixed effect model. We used this same linear mixed effect model approach to evaluate differences in *Fv/F_m_* values between drought deciduous and evergreen species at three temperatures: 24, 34 and 44°C. We ran additional models with these subsets of *Fv/F_m_* data with temperature, drought strategy and their interaction as fixed effects and species identity as a random effect. We then explored relationships between leaf thermotolerance values (*T*_Crit_, *T*_50_, and *T*_95_) and plant functional traits across all species using linear regression. We used the ‘*lm*’ function to run individual regression models with species’ means for each trait as the predictor and thermotolerance values as the response variable. Next, we used principal components analysis (PCA) to visualize and evaluate trait variation among plant species more holistically and to reduce dimensionality among these multiple, often covarying, traits to better characterize these differences and explore relationships with leaf thermotolerance. We conducted PCA using species’ mean trait values using the function ‘*prcomp*’. Values were standardized prior to ordination analysis. Following PCA, we explored correlations among traits and the first and second principal components (PC1 and PC2). Finally, we used linear regression to determine if these principal components representing overall trait variability among species explained levels of leaf thermotolerance.

## Results

### Drought deciduous species show lower leaf heat tolerance than evergreens

**Figure 3:**
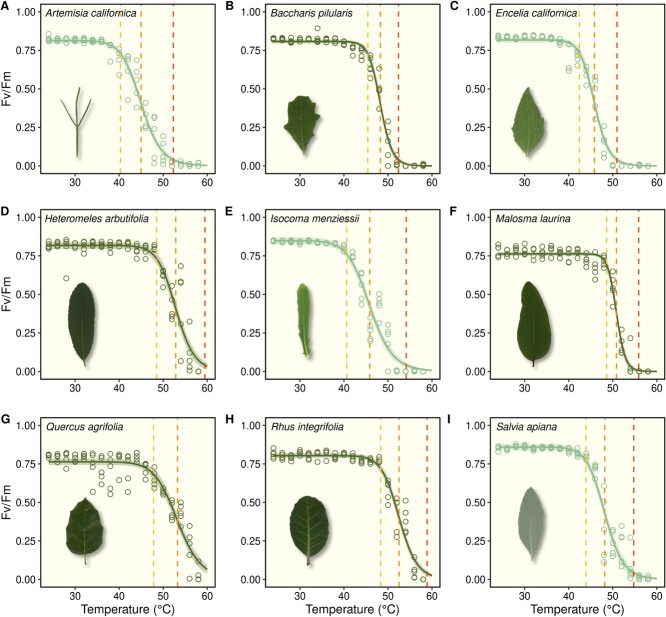
Temperature response curves for PSII functioning (*Fv/F_m_*) for each study species. Dashed lines indicate thresholds of *Fv/F_m_* decline, including the critical temperature (*T*_Crit_) at which photosynthetic function is irreversibly damaged (yellow), and the point at which *Fv/F_m_* is reduced to 50% (*T*_50_; orange) and 95% (*T*_95_; red) of maximum values observed.

Increasing temperatures had a negative effect on *Fv/F_m_* values for all species (Fig. 3). However, species showed substantial variation in leaf thermotolerance. Overall, drought deciduous species exhibited reductions in PSII functioning at lower temperatures compared with evergreen species. This greater leaf thermotolerance in evergreen species was further supported by the modeled temperature thresholds for loss of PSII function. Mean *T*_Crit_ estimates for drought deciduous and evergreen species were 42 and 48°C, respectively, while *T*_50_ estimates averaged 46°C for drought deciduous species and 52°C for evergreen species. At 24°C, evergreen species tended to have slightly lower *Fv/F_m_* values compared with drought deciduous species (*F_1,7_* = 11.26, *P* = 0.0121). However, at 34°C, *Fv/F_m_* values did not differ by drought strategy (*F_1,7_* = 3.22, *P* = 0.1158), and at 44°C, evergreen species exhibited significantly higher levels of PSII function than drought deciduous species. In the linear mixed effect model run on the subset of data from these three heat treatments, increasing temperature had a strong negative effect on *Fv/F_m_* values (*F_1,124_* = 98.33, *P* < 0.0001), but drought strategy alone had no significant effect (*F_1,7_* = 0.34, *P* = 0.5734). However, there was a highly significant interaction between temperature and drought strategy (*F_1,124_* = 41.77, *P ≤* 0.0001), indicating that these groups of species responded to the experimental heat treatments differently.

### Leaf functional traits vary by drought strategy

**Figure 4:**
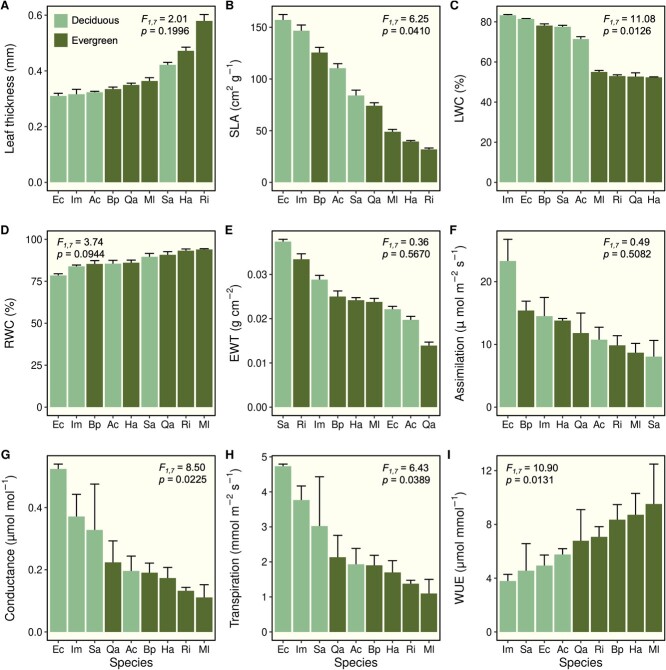
Mean morphological and physiological functional trait values across study species with different drought strategies (i.e. drought deciduous or evergreen), including leaf thickness (A), specific leaf area (B), leaf water content (C), relative leaf water content (D), equivalent water thickness of leaves (E), photosynthetic rates (F), stomatal conductance (G), transpiration (H) and instantaneous WUE (I). Error bars indicate standard error. Also shown are *F*-statistics and *P*-values from linear mixed effect models evaluating the effect of drought strategy on each functional trait.

**Figure 5:**
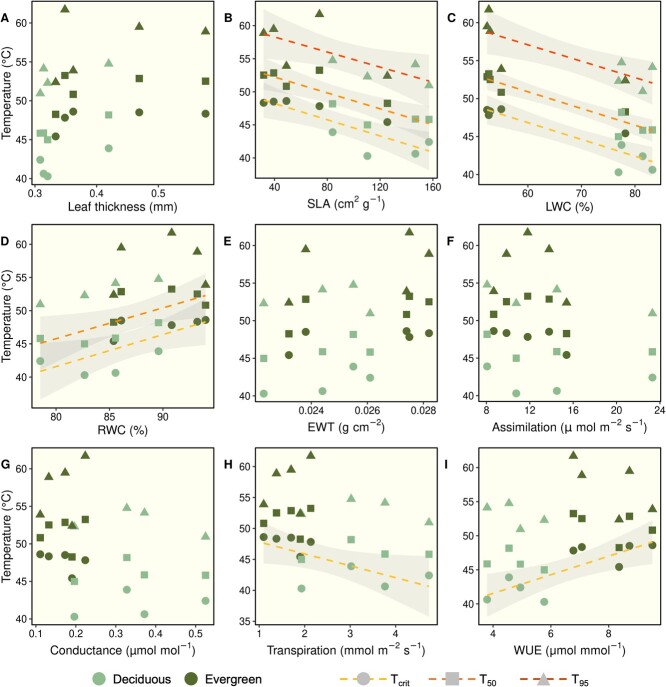
Scatterplots illustrating the influence of plant functional traits on thermotolerance values (*T*_Crit_, *T*_50_, and *T*_95_) across species. Only significant regression lines are shown. Line color and point shape denote temperature thresholds, while point color indicates species’ drought strategies. Predictors in regression models included leaf thickness (A), specific leaf area (B), leaf water content (C), relative leaf water content (D), equivalent water thickness of leaves (E), photosynthetic rates (F), stomatal conductance (G), transpiration (H) and instantaneous WUE (I).

Species exhibited a wide range of morphological and physiological functional trait values (Fig. 4 & 5), and many of these functional traits differed depending on species’ particular drought strategy. Leaf thickness tended to be higher in evergreen species with sclerophyllous leaves, but this did not differ significantly by drought strategy (Fig. 4A). However, leaf area per unit mass (SLA) was significantly greater in drought deciduous species as compared with evergreen species (Fig. 4B). As a whole, leaves of drought deciduous species had significantly greater water content (LWC) compared with evergreen species (Fig. 4C), while evergreen species maintained LWC closer to their maximum water holding capacities (i.e. greater RWC; Fig. 4D). Equivalent water thickness of leaves did not differ significantly by drought strategy (Fig. 4E). While we did not observe significant differences in rates of photosynthesis (Fig. 4F), these two groups of species exhibited distinct water use strategies, with drought deciduous species exhibiting greater stomatal conductance (Fig. 4G) and rates of transpiration (Fig. 4H) and markedly lower levels of WUE_i_ (Fig. 4I) compared with evergreens.

### Functional traits predict leaf thermotolerance

Linear regression models revealed several significant relationships (Table 2, Fig. 5) between species’ mean trait values and levels of leaf thermotolerance for *Fv/F_m_* (i.e. *T*_Crit_, *T*_50_ and *T*_95_). Thermotolerance thresholds tended to be higher for thick-leaved species, but leaf thickness had no effect on *T*_Crit_, *T*_50_ and *T*_95_ values in linear regression models (Fig. 5A). We detected a significant negative relationship between both SLA (Fig. 5B) and LWC (Fig. 5C) and *T*_Crit_, *T*_50_ and *T*_95_ values across species. These patterns were driven by substantially lower levels of leaf thermotolerance in drought deciduous species with high SLA and LWC values and compared with evergreen species. Leaf RWC showed the opposite pattern; greater RWC was associated with greater *T*_Crit_ and *T*_50_ values across species, with evergreen species exhibiting higher values for each of these variables (Fig. 5D). There were no significant relationships between leaf EWT (Fig. 5E) or photosynthetic rates (Fig. 5F) with leaf thermotolerance values across species. We observed an overall negative relationship between stomatal conductance and *T*_Crit_ and *T*_50_ values (Fig. 5G), but these were not significant (Table 2). Thermotolerance levels also tended to be lower among species with greater rates of transpiration (Fig. 5H) and lower WUE_i_ (Fig. 5I), but these relationships were only significant for *T*_Crit_ values (Table 2).

**Table 2:** Results of linear regressions evaluating the relationship between mean trait values and thermal tolerance thresholds across species. Traits include leaf thickness (Thick), specific leaf area (SLA), leaf water content (LWC), relative water content (RWC), equivalent water thickness (EWT), photosynthetic rate (Photo), stomatal conductance (g_s_), transpiration (E) and instantaneous water use efficiency (WUE_i_)

	T_crit_		T_50_		T_95_	
Trait	*R^2^*	*p*	*R^2^*	*p*	*R^2^*	*p*
Thick	0.37	0.0806	0.43	0.0560	0.35	0.0960
SLA	**0.70**	**0.0052**	**0.73**	**0.0035**	**0.50**	**0.0326**
LWC	**0.81**	**0.0009**	**0.88**	**0.0002**	**0.65**	**0.0085**
RWC	**0.50**	**0.0317**	**0.51**	**0.0297**	0.34	0.1002
EWT	0.33	0.1028	0.33	0.1076	0.20	0.2282
Photo	0.11	0.3775	0.15	0.3064	0.15	0.3063
g_s_	0.41	0.0630	0.37	0.0803	0.19	0.2343
E	**0.45**	**0.0476**	0.40	0.0687	0.19	0.2382
WUE_i_	**0.63**	**0.0102**	0.42	0.0608	0.08	0.4708

### Drought strategy predicts leaf thermotolerance

**Figure 6:**
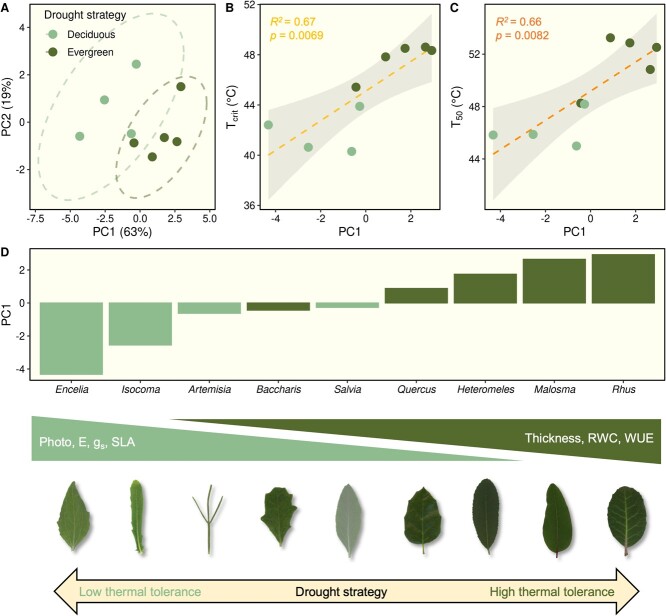
Ordination of species in trait space from principal components analysis of mean trait values across species (A) and linear regressions between species’ location along the first principal component (PC1) and *T*_crit_ (B) and *T*_50_ (C) values. Also shown are species’ scores for PC1 (D), which was strongly correlated with a number of leaf-level traits, and for which drought deciduous and evergreen species showed a marked contrast.

The ordination of study species in trait space (Fig. 6A) further illustrated differences in leaf morphology and water use between drought deciduous and evergreen species. Collectively, the first two principal components explained 82% of the variance among plant species. The first principal component (PC1) explained 62% of the variance, and it was along this axis that the two groups of species showed a clear separation (Fig 6A). PC1 was strongly correlated with a number of leaf-level traits, including (listed in order of decreasing importance) SLA (*r* = −0.95, *P* < 0.0001), transpiration (*r* = −0.93, *P* = 0.0003), stomatal conductance (*r* = −0.92, *P* = 0.0005), LWC (*r* = −0.86, *P* = 0.0029), RWC (*r* = 0.85, *P* = 0.0038), photosynthetic rate (*r* = −0.76, *P* = 0.0186), WUE (*r* = −0.76, *P* = 0.0186) and leaf thickness (*r* = 0.69, *P* = 0.0395). PC2 explained 19% of the variance in the trait dataset and was highly correlated with leaf EWT (*r* = −0.94, *P* < 0.0001). Given the strong explanatory power of PC1 in characterizing overall trait variation among species and distinguishing between drought deciduous and evergreen species, we were especially interested in evaluating if species’ location along this axis was related to levels of leaf thermotolerance. We found a significant positive relationship between species’ scores on PC1 and both *T*_Crit_ (Fig. 6B) and *T*_50_ (Fig. 6C) values. Evergreen species with thick leaves, high RWC and high WUE exhibited greater leaf thermotolerance and more positive scores for PC1, while drought deciduous species were located on the opposite end of this axis, which was associated with greater SLA and LWC, as well as higher rates of carbon assimilation, transpiration and stomatal conductance (Fig. 6D).

## Discussion

Heat stress imposes an important physiological constraint on native plant species—one that will only worsen with human-caused climate change (Anderson, 2016; O'Sullivan *et al.,* 2017; McDowell *et al.,* 2022). Understanding how plant species will respond physiologically to rising temperatures and how these responses differ among plant functional groups is critical for predicting future biodiversity scenarios (Soudzilovskaia *et al.,* 2013; Urban *et al.,* 2016) and making informed land management decisions ([Bibr ref12][Bibr ref12]; Tomlinson *et al.,* 2022; Valliere *et al.*, 2022). The results of this study highlight the amount of functional trait diversity that exists among native plant species of southern California, which allows them to withstand periods of prolonged hot and dry conditions. Importantly, we show that these species’ drought strategies—and the traits that underlie them—are strong predictors of leaf heat tolerance.

### Drought avoidance and tolerance: Two different solutions to the same problem

Our results are consistent with what has been previously demonstrated in a large body of research: perennial plant species from Mediterranean-type ecosystems that experience seasonal drought have evolved contrasting water use strategies and leaf-level functional traits (Harrison *et al.,* 1971; Poole and Miller, 1975; Ackerly, 2004; Pivovaroff *et al.,* 2018). Drought deciduous species of California’s coastal sage scrub possess leaf traits associated with rapid return on leaf investments, including high rates of gas exchange, high SLA and low WUE. As our results show, these species utilize water quickly when soil moisture availability is adequate and therefore exhibit high rates of transpiration, low leaf RWC and low WUE. These traits enable rapid carbon assimilation when conditions are favorable but are not well suited for periods of water limitation. Therefore, these species avoid drought conditions (and high temperatures) by dropping their leaves when soil moisture is depleted. In contrast, the evergreen trees and shrubs investigated here produce more costly, denser and thicker sclerophyllous leaves that may have slower returns in terms of carbon assimilation, but their more conservative water use allows them to persist during periods of sustained drought while maintaining physiological activity. It should be noted, however, that these strategies are not a simple binary. Instead, species exhibited functional trait values along a continuum that aligns with the leaf economic spectrum (Wright *et al.,* 2004); drought deciduous species possess leaf traits associated with rapid resource use and C gain, while evergreen species possess leaf traits associated with resource conservation. In addition to differences due to water use strategies, much of the observed trait variation among species is also likely related to leaf longevity (Reich *et al.,* 1992), with drought deciduous species constructing relatively “cheap”, short-lived leaves and evergreen species investing greater construction costs for leaves that persist much longer (Gray, 1982). These trait differences related to water use and leaf longevity also appear to play an important role in leaf heat tolerance.

### Leaf heat tolerance is predicted by functional traits and drought strategy

Overall, we found that drought deciduous species experienced a loss of photosynthetic functioning at lower temperatures than evergreen species. Furthermore, several leaf-level functional traits were found to be significant predictors of leaf thermotolerance; drought deciduous species with high SLA, LWC and transpiration, and low RWC and WUE were the most susceptible to heat damage. Conversely, sclerophyllous species with thicker and denser leaves, high RWC, low rates of transpiration and high WUE maintained photosynthetic function at higher temperatures. These functional trait differences and their potential role in driving leaf thermotolerance were further illustrated by the results of our ordination analysis. This yielded a principal component that represented the well-established trade-off between resource acquisition and investment in leaf construction costs (Wright *et al.,* 2004), with drought deciduous and evergreen species occupying opposite ends of this spectrum. Furthermore, the position of species along this axis was a strong indicator of observed thermotolerance thresholds. Recent research in other ecosystems has identified similar relationships between leaf functional traits and thermotolerance, where species that produce leaves with lower mass per area are more susceptible to heat stress than species with relatively thicker and more dense leaves (Sastry and Barua, 2017; Sastry *et al.,* 2018; Slot *et al.,* 2021). Our results show that similar mechanisms may mediate leaf heat tolerance in plants from semi-arid Mediterranean regions, and SLA was identified as a strong predictor of thermotolerance thresholds in our species. However, unlike this previous work, we also show that several leaf traits related to water use, including LWC, RWC and WUE, appeared to explain variable levels of leaf heat tolerance across species.

As a whole, the seasonally deciduous species exhibited lower levels of leaf thermotolerance, which could make them more vulnerable to rising temperatures and heat wave events. While these drought-avoidant species are able to escape the greatest risk of heat stress that occurs during summers, extreme heat days during the winter and spring growing season could have severe consequences for their performance and survival. The thermotolerance thresholds of evergreens species are, for the most part, above predicted maximum air temperatures for the region. However, these projections do not take into account the influence of urban heat islands or the fact that leaf temperatures can far exceed that of surrounding air (O'Sullivan *et al.,* 2017), especially if plants cease transpiring. For example, Valladares and Pearcy (1997) recorded leaf temperatures in *Heteromeles arbutifolia* that were up to 10°C above ambient temperatures, and in some instances exceeded 50°C—a temperature greater than the point at which all species included in this study exhibited irreversible damage to photosynthetic capacity. Furthermore, while evergreen species may possess adaptations for greater leaf heat tolerance compared with drought deciduous species, their year-round canopy cover means they experience the much greater leaf temperatures that occur during summer. These high summer temperatures also coincide with periods of low soil water availability when transpirational cooling could be quite limited. Thus, even these more heat-tolerant species could experience heat-induced disruption in photosynthetic activity as well as more severe consequences for plant functioning and health. Nevertheless, our work is consistent with previous work (Knight and Ackerly, 2003; Sastry *et al.,* 2018; Marchin *et al.,* 2022) showing that species with leaf-level traits associated with less conservative water use are more susceptible to heat stress, which threatens the persistence of these species in urban areas. For example, Marchin *et al.* (2022) compared crown dieback and mortality in urban forests of Sydney, Australia, during the hottest summer on record in 2019. They related species’ responses to extreme temperatures to leaf functional traits, finding that native drought-avoidant species with similar leaf traits to the coastal sage scrub species in our study were substantially more vulnerable than evergreen species with more drought-tolerant leaf traits (Marchin *et al.,* 2022).

### Limitations and future directions

We included a variety of species that exhibit a wide range of leaf trait values, which allowed us to identify key relationships between leaf morphology, water use and leaf thermotolerance. However, our species selection was limited by the availability of native species in the restored urban nature preserve that served as our study site. Further investigations that include more species within the region (and in other Mediterranean-type ecosystems worldwide) as well as populations of the same species across climate gradients would be insightful. It should also be noted that we sampled leaves during a period of high rainfall in southern California, and therefore plants were under relatively low levels of stress. Water limitation can have an important influence on heat tolerance in plants, and understanding how the combined effects of drought and increased temperature influences these species is an important avenue of future research (López *et al.,* 2022). It would also be useful to evaluate other traits and biochemical mechanisms that underlie the observed responses among these species, such as differential expression of heat shock proteins (Knight, 2010). Species may also differ in their capacity to acclimate to heat stress (Sastry and Barua, 2017), and comparisons among species under different weather conditions throughout the year could shed light on the relative importance of these plastic responses in mediating heat tolerance. For example, many drought deciduous species in southern California exhibit seasonally dimorphic leaves (Westman, 1981), and it would be interesting to evaluate if the observed relationships between leaf functional traits and thermotolerance also operate within individual species across seasons. Finally, measurements of leaf temperatures along with transpiration rates in the field would also be useful for explaining observed differences between species. For example, species with lower transpiration rates (e.g. the evergreen species explored here) could experience greater leaf temperatures, thereby necessitating other mechanisms for leaf thermotolerance.

### Guiding plant conservation in a warmer world

Identifying the thermal limits of plant species is essential for understanding vulnerabilities in a warming world, but this knowledge can also be used to directly guide conservation and restoration strategies (Tomlinson *et al.,* 2022; Valliere *et al.,* 2022). Specifically, information on plant heat tolerance could guide the preparation and selection of plant material used in revegetation efforts (Rice and Emery, 2003; Valliere *et al.,* 2022). For example, species with greater leaf heat tolerance may be more appropriate for restoration projects and urban plantings in areas expected to experience increased temperatures, and the approach outlined in this paper can guide such selections. Species from hotter climates (Knight and Ackerly, 2002) and even warmer microclimates within the same region (Knight, 2010) may also possess a greater capacity to withstand higher temperatures, and the methods used here could be applied to evaluate this to guide conservation efforts. Plants may also exhibit greater stress tolerance due to acclimation responses (Valladares and Pearcy, 1997; Valliere *et al.,* 2019; Grossman, 2023), and practitioners could take advantage of this plasticity to improve restoration outcomes in the context of climate warming. For example, Valliere *et al.* (2019) found that severe drought treatments applied to seedlings of native perennials in southern California resulted in greater SLA and WUE (and consequently greater restoration success), and these effects lasted months after the initial treatment. Based on the results of this study, such leaf-level adjustments in morphology and physiology could also yield greater thermotolerance. Finally, the integration of thermotolerance thresholds with species distribution modeling represents a promising approach for modeling how species’ ranges may be impacted by projected increases in temperatures and identify those that are the most vulnerable.

## Conclusions

Restored native plant communities and native plant gardens—such as the ones utilized in this study—represent an important strategy for conserving biodiversity in urban areas (Dearborn and Kark, 2010; Segar *et al.,* 2022). They may also play a critical role in directly combatting the urban heat island effect and aid cities in meeting climate adaptation strategies (Butt *et al.,* 2018; Espeland and Kettenring, 2018). However, our work highlights how projected climate change could hamper such efforts if species are unable to cope with new temperature extremes. The species included in this study belong to several imperiled plant communities in California that are threatened by human activities; in addition to rising temperatures, other drivers of environmental change including drought, air pollution, invasion and novel pests and pathogens pose a significant risk to coastal sage scrub (Valliere *et al.,* 2017; Valliere *et al.,* 2020), chaparral (Rundel, 2018; Syphard *et al.,* 2019), and oak woodlands (Rizzo *et al.,* 2002; Coleman *et al.,* 2011). Unless appropriate interventions are implemented to address these threats and preserve these species, their future remains uncertain in a hotter, increasingly urbanized California.

## Ackowledgements

We thank Gretchen North and Jochen Schenk for useful advice and discussions on methodology and Deepak Barua for sharing code for plotting and data analysis. We also thank Wallace Meyer, Nina Karnovsky and the RESCUE-NET team for their support of this work.

## Author Contributions

J.M.V. conceived of the research. K.C.N. conducted thermal tolerance assays and collected leaf trait data. M.C.M. collected plant gas exchange data. J.M.V., K.C.N. and M.C.M. analysed the data. J.M.V. prepared the figures and the manuscript. All authors contributed to editing.

## Conflicts of Interest

The authors have no conflicts of interest to declare.

## Funding

This work was supported by a grant from the California Attorney General’s Automobile Emissions Research and Technology Fund; the California Education Learning Lab; the CSU Louis Stokes Alliance for Minority Participation; and support from the National Science Foundation (Award # 2217253).

## Data Availability Statement

Data presented are archived in the online Dryad Data Repository (https://doi.org/10.5061/dryad.xsj3tx9n7).
